# Red ginseng prevents niraparib-induced myelosuppression in C57BL/6 mice via inhibiting p53-mediated upregulation of p21 and p27

**DOI:** 10.1007/s11418-024-01866-3

**Published:** 2024-12-30

**Authors:** Huiyan Liao, Xiangdan Hu, Shenming Chen, Zhaofeng Fan, Jing Xiao

**Affiliations:** 1 Guangdong Provincial Key Laboratory of Major Obstetric Diseases, Guangdong Provincial Clinical Research Center for Obstetrics and Gynecology, The Third Affiliated Hospital of Guangzhou Medical University, Guangzhou, Guangdong 510140 People’s Republic of China; 2 The Second Clinical College of Guangzhou, University of Chinese Medicine, Guangzhou, Guangdong 510006 People’s Republic of China; 3 Department of Gynecologic Oncology, the Second Affiliated Hospital of Guangzhou University of Chinese Medicine, Guangzhou, Guangdong 510120 People’s Republic of China

**Keywords:** Hematopoietic function, Bone marrow, Red ginseng, Niraparib, Mutant tumor protein 53

## Abstract

Myelosuppression is a serious and common complication of targeted therapy for cancer patients, and there are few studies exploring the efficacy of natural drugs in this condition. Niraparib is a widely used targeted therapy for the treatment of advanced ovarian cancer. As a poly (ADP-ribose) polymerase (PARP) inhibitor, niraparib significantly improves progression-free and overall survival in patients. We aimed to explore the potential effect of red ginseng (RG) on niraparib-induced myelosuppression and to further reveal its possible molecular mechanism. Female C57BL/6 mice were divided into control, tumor, model, and RG groups (n = 6). After receiving ID8 ovarian cancer cell inoculation, the mice received niraparib treatment (80 mg/kg) for 3 days. Meanwhile, RG groups (100 and 200 mg/kg) were intragastrically treated with RG extract for 7 days. Compared with the model group, RG extract increased the counts of peripheral blood cells and enhanced the hematopoietic function of bone marrow. Furthermore, RG extract increased the colony yield of hematopoietic progenitor cells (HPCs), facilitated DNA damage repair, alleviated the G0/G1 phase cell cycle arrest, and significantly reversed the increased expression levels of p53, p21, and p27, while stimulating cyclinE1 expression levels. These findings indicate that RG might have therapeutic potential on niraparib-induced myelosuppression, which encourages further clinical trials. This study is the first to explore the efficacy and mechanism of RG in preventing myelosuppression induced by niraparib.

**Graphical abstract:**

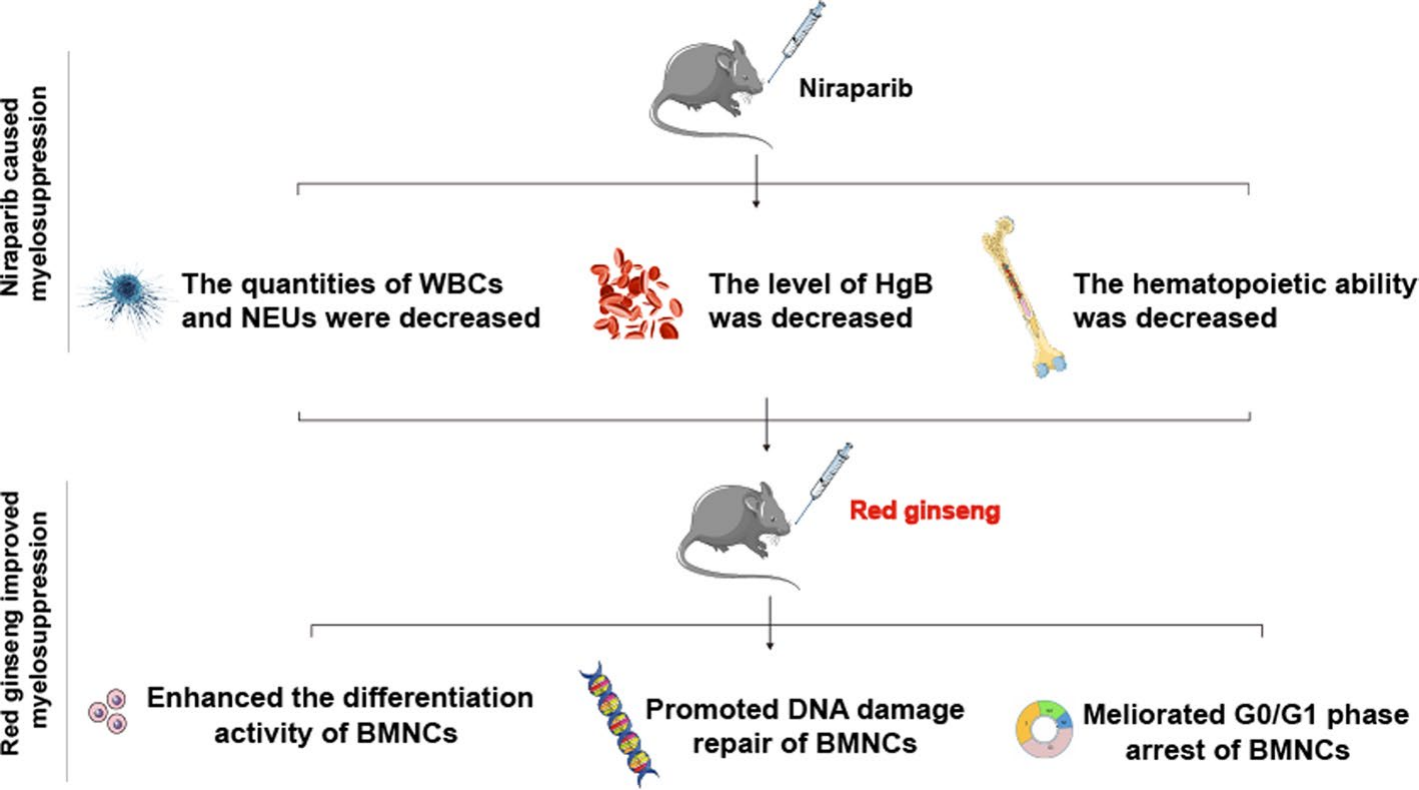

## Introduction

Niraparib is a widely used targeted therapy for the treatment of advanced ovarian cancer. As a poly (ADP-ribose) polymerase (PARP) inhibitor, niraparib significantly improves progression-free and overall survival in patients [1]. However, PARP in the bone marrow may also be inhibited by PARP inhibitors, leading to myelosuppression [2, 3]. Severe myelosuppression can lead to drug interruption or discontinuation, thus affecting the patients’ quality of life and survival benefits.

Red ginseng (RG) is a processed product of *Panax ginseng* C.A. Meyer (Araliaceae) prepared by repeated steaming and drying of fresh ginseng [4]. It has been consumed as a dietary supplement and herbal medicine in East Asia for thousands of years. RG and its components can help alleviate chemotherapy-induced myelosuppression. In mice with 5 fluorouracil-induced myelosuppression, RG increases peripheral cell counts, the number of bone marrow colony-forming unit granulocytes and macrophages (CFU-GM), and granulocyte–macrophage colony-stimulating factor (GM-CSF) [5]. In a cyclophosphamide (CTX)-induced myelosuppression model, RG alleviated myelosuppression by increasing the yields of CFU-GM, colony-forming unit-granulocyte, -erythrocyte, -monocyte, -megakaryocyte (CFU-GEMM), burst-forming unit-erythroid (BFU-E), and colony-stimulating factors [6]. However, the molecular mechanisms underlying the action of RG on targeted drug-induced myelosuppression remain unknown.

We, therefore, aimed to explore the potential effect of RG on niraparib-induced myelosuppression and to further reveal its possible molecular mechanism. To the best of our knowledge, this is the first study to examine the effect of RG on niraparib-induced myelosuppression.

## Methods

### Materials and reagents

The RG, grown in Jilin Province, China, was purchased from Kangmei Xinkaihe Pharmaceutical Co., Ltd. in 2020. Its quality complied with the Chinese Pharmacopoeia (Version 2020). A voucher specimen (No. 200302) was deposited for reference in the Guangdong Branch of the Chinese Academy of Traditional Chinese Medicine. The sample was stored in the shade at room temperature before use. Niraparib tosylate (NO. 522106003) powder was obtained from Zai Lab Co. Ltd. (Shanghai, China). Mouse granulocyte–macrophage colony-stimulating factor (GM-CSF) (315-03-5), erythropoietin (EPO) (100-64-10), and interleukin-3 (IL-3) (213-13-2) were supplied by PeproTech Inc. (Suzhou, China). Rabbit monoclonal anti-p53 (2527) and anti-p27 (3688) antibodies were purchased from Cell Signaling Technology (Danvers, MA). Rabbit monoclonal anti-γ-H2AX (ab81299) antibody was purchased from Abcam (Cambridge, UK). Rabbit monoclonal anti-p21 (27296-1-AP), anti-Ki-67 (28074-1-AP), and anti-CyclinE1 (11554-1-AP) were purchased from ProteinTech (Wuhan, China). Dulbecco’s modified Eagle’s medium (DMEM) and fetal bovine serum (FBS) were acquired from Gibco (Shanghai, China). All other chemicals were of the highest grade and purchased from commercial sources.

### Cell line

ID8, a murine ovarian cancer cell line, was obtained from College of Life and Technology, Jinan University. The cell line was cultivated in DMEM with 10% FBS, 100 U/mL penicillin, and 100 μg/mL streptomycin. The cell line was maintained at 37 °C with 5% CO_2_ in a humidified incubator.

### UHPLC-QE-MS analysis of RG extracts

Ginseng slices (100 g) with a thickness of approximately 0.3 cm were weighed and extracted twice for 1.5 h with tenfold (w/v) water at 100 °C. The extract was concentrated to 250 mg of dried crude herb per milliliter. The constituents of RG and reference substances were identified using ultra-performance liquid chromatography (UPLC) in conjunction with electrospray ionization mass spectrometry (ESI–MS). Samples were separated on a Thermo Fisher Scientific Hypersil GOLD (100 × 2.1 mm, 1.9 μm) at 40 °C. The mobile phase was composed of A (0.1% formic acid in water) and B (acetonitrile) with a gradient program: 0–3 min, 10% B–20% B; 3–25 min, 20% B–38% B; 25–30 min, 38% B-85% B; 30–30.1 min, 85% B–5% B; 30.1–32 min, 5% B. The flow rate was set to 0.3 mL/min. The sample solution (5 μL) was injected for each run. The MS analysis was performed using a Thermo Scientific Q Exactive Series (Thermo Fisher Scientific, USA). The mass spectrometer was connected to the UPLC system using a heated electrospray ionization source and operated in both positive and negative ion modes. Data were collected at *m*/*z* 100–1500. The MS full scan was detected by high-resolution (FT, R = 70,000) and MS/MS analysis by resolution (FT, R = 17,500). The optimized source parameters in positive (and negative) modes were as follows: capillary temperature, 320 °C; sheath gas flow, 45 arb.; auxiliary gas flow, 10 arb.; spray voltage, 3500 V. The data were collected and processed using the Xcalibur 2.1 software (Thermo Scientific).

### Animal experiments

Female C57BL/6 mice, with body weights ranging from 16 to 18 g, were obtained from the Medical Laboratory Animal Center of Guangdong Province (Certificate No. 044002000204). The mice were housed under standard controllable experimental conditions with a temperature of 22 ± 2 °C, relative humidity of 50 ± 10%, and a 12-h light/dark cycle. All efforts were made to minimize pain in the animals. Animal procedures were performed in accordance with the National Institute of Health Guide for the Care and Use of Laboratory Animals and approved by the Animal Ethics Committee of Guangdong Academy of Traditional Chinese Medicine (No. 2020069). After 1 week of acclimatization, the mice were divided into five groups (n = 12): normal (control), tumor (tumor), niraparib (model), RG low-dose (RG-L, 100 mg/kg), and RG high-dose (RG-H, 200 mg/kg).

A 1 × 10^7^ ID8 cell line was subcutaneously inoculated into the right flanks of the mice in the tumor, model, RG-L, and RG-H groups. Tumor size was measured with digital calipers every 3 days and the animals were administrated 21 days after inoculation. RG was continuously administered for 14 days to the RG-L (100 mg/kg) and RG-H (200 mg/kg) groups. The model, RG-L, and RG-H groups were intragastrically administered a single dose of niraparib solution (80 mg/kg) in the morning from day 5 to day 7, while the control and tumor group were treated with an equal volume of saline on the same schedule. On day 8, 24 h after niraparib treatment, six mice were randomly selected from each group and given adequate anesthesia. Blood samples, femurs, and tibias were collected from each group to estimate peripheral blood cells and bone marrow cells. On day 15, all remaining mice were anesthetized adequately and dissected. Tumor tissues were collected, measured in size, and immunohistochemical analyses were performed.

### Detection of peripheral blood cells and bone marrow nucleated cells (BMNC)

Blood samples from the eye socket veins of each group were collected in anticoagulant tubes containing Ethylenediaminetetraacetic acid (EDTA). White blood cells (WBCs), neutrophil granulocytes (NEUs), and platelets (PLTs) were counted and hemoglobin (HgB) content was determined. After blood samples were collected, mice were euthanized and placed in 75% ethanol for 5 min twice. Both femurs and tibias were removed under aseptic conditions, and bone marrow cells were flushed using Iscove’s modified Dulbecco’s medium (IMDM) to form a single-cell suspension. After the erythrocytes were lysed, the remaining BMNC were rinsed twice with 2 mL of sterile phosphate buffered solution (PBS) and counted.

### Hematopoietic progenitor cells (HPCs) culture

BMNCs were plated at a concentration of 1 × 10^5^/mL in IMDM medium supplemented with FBS, 10^–4^ mol/mL β-mercapto ethanol, 3% l-glutamine, 20 IU/mL EPO, 50 ng/mL GM-CSF, and 20 ng/mL interleukin-3. Cells were cultured at 37°C in a 5% CO_2_ atmosphere. CFU-E were counted under an inverted phase-contrast microscope after 3 days of culture. The BFU-E and the CFU-GM were counted after 7 days of culture.

### Hematoxylin and eosin (H&E) staining

The mouse sternums were harvested and fixed in 4% paraformaldehyde immediately. They were decalcified using formic acid, embedded in paraffin, and sectioned into 4-µm sections before H&E staining. Histological images were acquired using a light microscope (Nikon).

### Cell cycle

For each group, 1 × 10^6^ BMNCs were collected in a centrifuge tube and rinsed twice with PBS. Cells were incubated with 1 mL DNA staining solution and 10 µL permeabilization solution in the dark for 30 min at room temperature (RT). Cells from all groups were counted using flow cytometry at low speed. The proliferation index (PI) was calculated using the following formula: PI = (S + G2/M) × 100%/(G0/G1 + S + G2/M).

### Western blotting of bone marrow cells

Total protein in the BMNCs was extracted, and protein concentrations were determined using a multi-function microplate reader. The protein samples were denatured and separated by sodium dodecyl sulfate polyacrylamide gel electrophoresis (SDS-PAGE) electrophoresis, transferred to poly vinylidene fluoride membranes by semi-dry electrophoresis, and blocked using 5% skimmed milk liquid for 2 h. Primary antibodies were added and incubated at 4 °C overnight, and a secondary antibody was added and incubated for 1.5 h at RT. β-Actin was used as an internal reference. After the film was scanned, the protein bands were quantitatively analyzed and the gray ratios of the target protein band and internal reference band were calculated as the relative expression level of each target protein.

### γ-H2AX immunofluorescence

γ-H2AX is a specific marker of double-strand breaks (DSBs). BMNCs were cytospun onto glass slides and fixed with 4% paraformaldehyde for 10 min at RT. After permeabilization and blocking, the fixed cells were incubated with the anti-γ-H2AX antibody at a dilution of 1:250 at 4 °C overnight. Cells were then incubated with Alexa 488-conjugated goat anti-rabbit γ-H2AX at RT for 1.5 h. The slides were consecutively washed 3 times and stained with 4′,6-diamidino-2-phenylindole (DAPI) for 8 min at RT. The slides were subsequently washed 3 times and mounted with an anti-fade solution. Images were captured using an inverted microscope (Olympus, Tokyo, Japan).

### Immunohistochemical analysis

Paraffinized tumor sections were used for immunohistochemistry (IHC) to target Ki-67. First, paraffin sections were baked at 60°C overnight, deparaffinized with xylene, and rehydrated with ethanol. Sodium citrate antigen retrieval solution was used for antigen retrieval in a microwave. Hydrogen peroxide was used to block endogenous peroxidase activity, and a protein-blocking reagent was used to block irrelevant antigens. The sections were incubated with the primary Ki-67 antibody (1:250) overnight at 4°C. After adding the secondary antibodies, the slides were stained with Strept Avidin–Biotin Complex and 3,3′-diaminobenzidine (DAB). Hematoxylin was used for counterstaining, and the slides were dehydrated.

### Statistical analysis

All data are expressed as mean ± standard deviation (SD). Statistical analyses were performed using an independent sample *t*-test and one-way analysis of variance using SPSS software (version 26.0; SPSS, Chicago, IL). A *p* value < 0.05 was considered statistically significant.

## Results

### Composition of RG determined by UHPLC-QE-MS

**Fig. 1:**
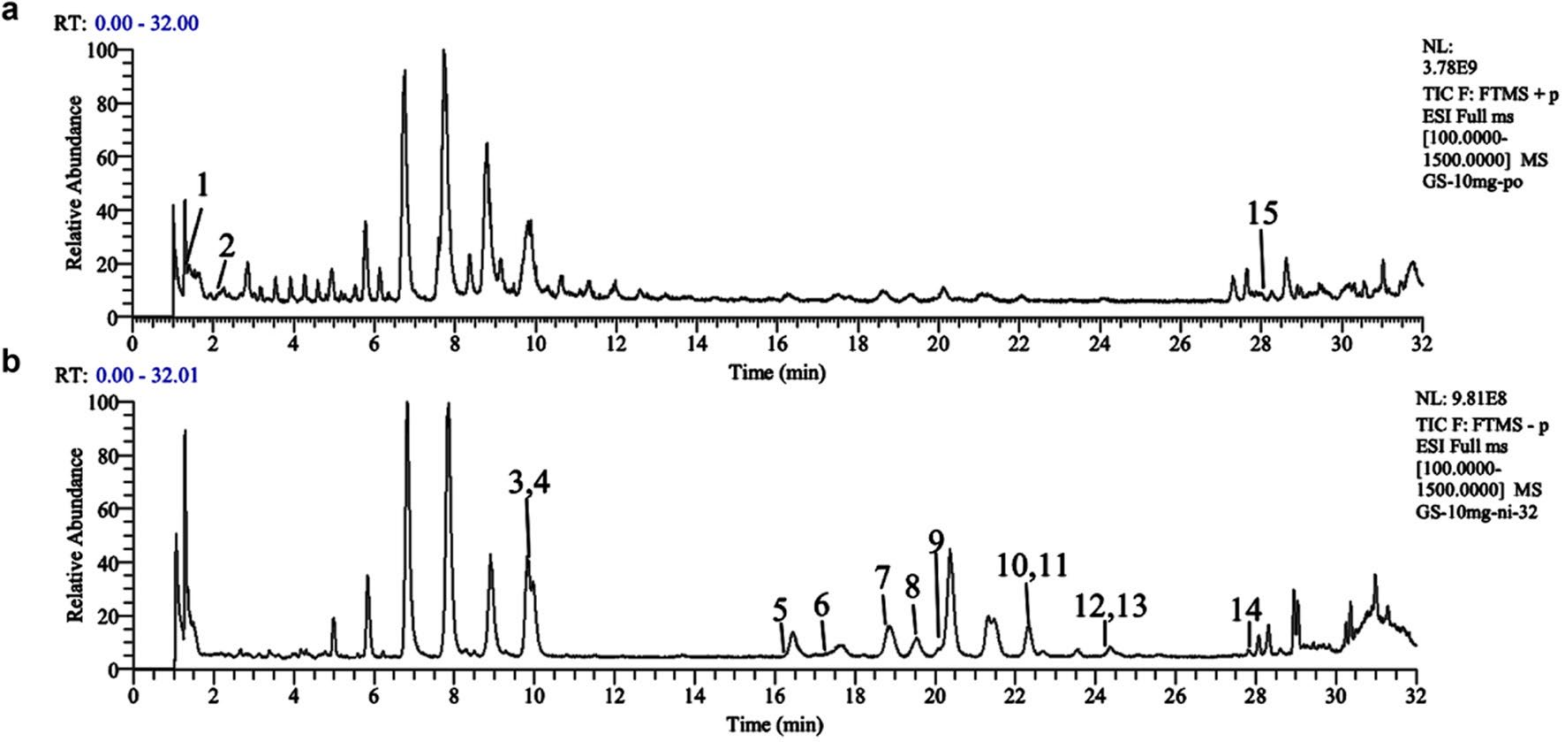
The TIC of RG was determined by UHPLC-QE-MS analysis. TIC of RG in positive (**a**) and negative (**b**) ion modes

RG solution was prepared at a concentration of 10 mg/mL in 70% methanol and stored at 4°C until use. The RG solution was analyzed using UHPLC-QE-MS, and both positive and negative ion modes were used to scan the RG solution. Figure 1 displays the total ion current (TIC) obtained from the analysis. Nine compounds were tentatively characterized and unambiguously identified by comparison with reference standards [7]. For other compounds, the structures were tentatively identified by referring to previous studies and considering factors such as accurate mass, MS/MS data, fragmentation rules, and chromatographic behavior [8]. Table 1 shows the retention time, mass spectrometry information, and structural formula for each of the chemical components.

**Table 1:**
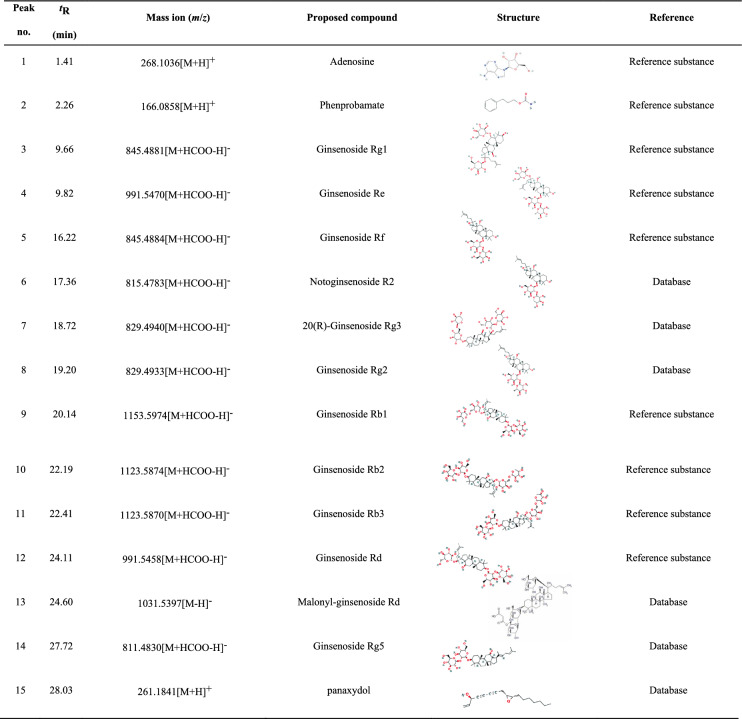
Information on natural chemical products in RG

### Effect of RG on peripheral blood cells and bone marrow

**Fig. 2:**
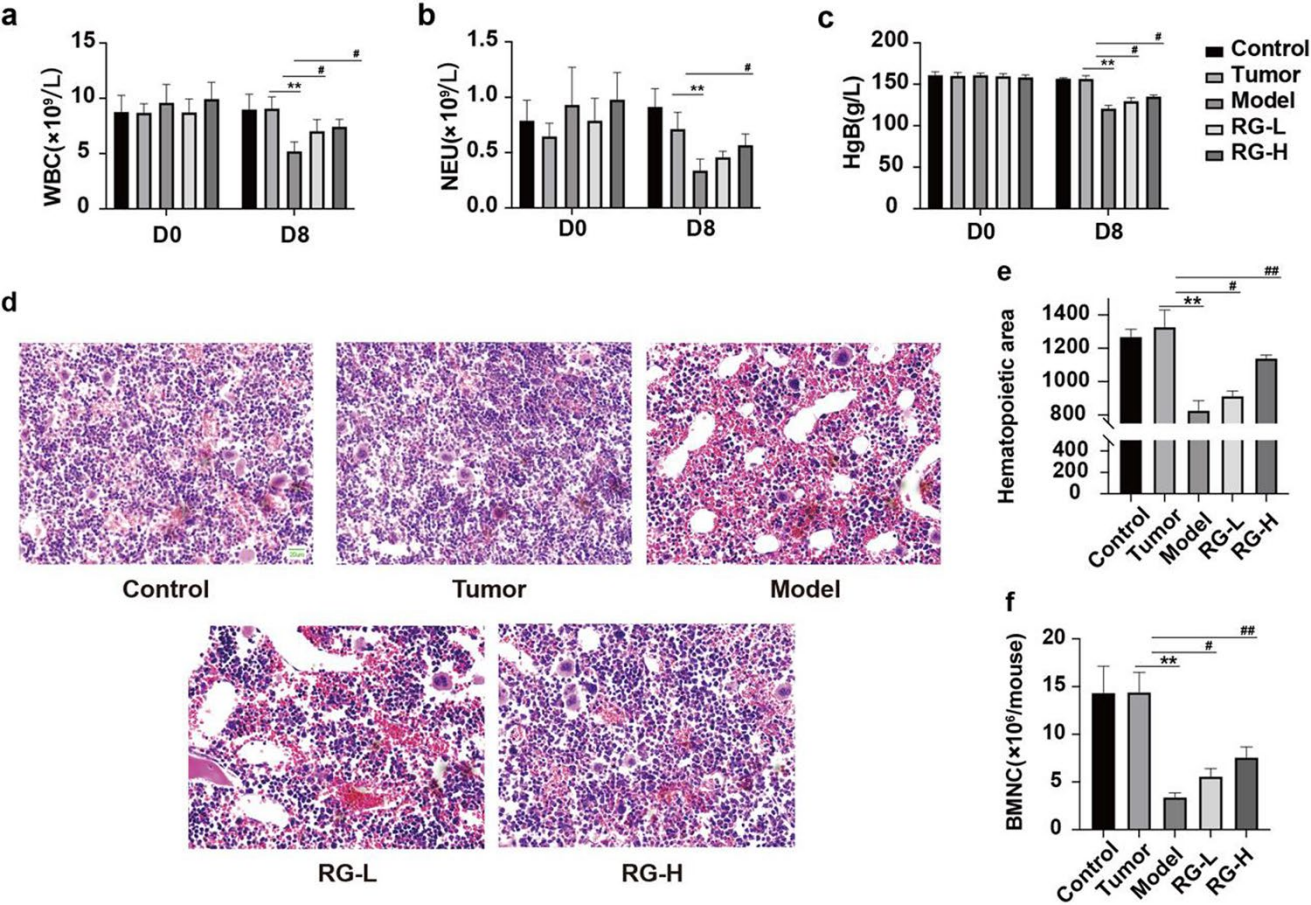
Effect of RG on the hematopoietic function of niraparib-induced mice. The counts of WBC (**a**), NEU (**b**), and HgB (**c**) in the peripheral blood. Values are expressed as mean ± SD (n = 6). Statistical significance was taken at **p < 0.01 compared with the tumor group, ^#^*p* < 0.05 compared with the model group. The bone marrow tissue histomorphology of the sternum (**d**) and the hematopoietic area thereof (**e**); the counts of bone marrow nucleated cells (**f**) in the bone marrow of both femurs and tibias. Values are expressed as mean ± SD (n = 3). Statistical significance was taken at **p < 0.01 compared with the tumor group, ^#^*p* < 0.05, ^##^*p* < 0.01 compared with the model group. Sections were observed at ×400. Scale bar indicated in the control group is 20 µm

Myelosuppression is a state of decreased bone marrow activity, resulting in a decrease in red blood cells (RBCs), white blood cells (WBCs), and platelets (PLTs) [9]. Therefore, we investigated the protective effects of RG on peripheral blood cell and bone marrow hematopoietic area levels. As shown in Fig. 2a–c, there were no significant differences in WBC, neutrophil (NEU), and hemoglobin (HgB) counts between the control and the tumor groups, suggesting that the presence of a tumor might not have a considerable effect on hematopoietic function in mice. Niraparib significantly reduced WBC, NEU, and HgB counts, and both RG-L and RG-H treatments could significantly increase the quantities of WBCs and HgB. Only the RG-H treatment significantly increased the quantity of NEU.

As shown in Fig. 2d–f, there were still no significant differences in the quantity of BMNCs and hematopoietic area between the control and the tumor groups. After modeling, the BMNCs were loosely arranged, the fat area increased, and the quantity of BMNCs were also significantly decreased, leading to a diminished hematopoietic area. As expected, both RG-L and RG-H treatments significantly increased the hematopoietic area and the quantity of BMNCs. The results indicated that RG remarkably improved the hematopoietic function of mice.

### *Effect of RG on the colony yield of HPCs *in vitro

**Fig. 3:**
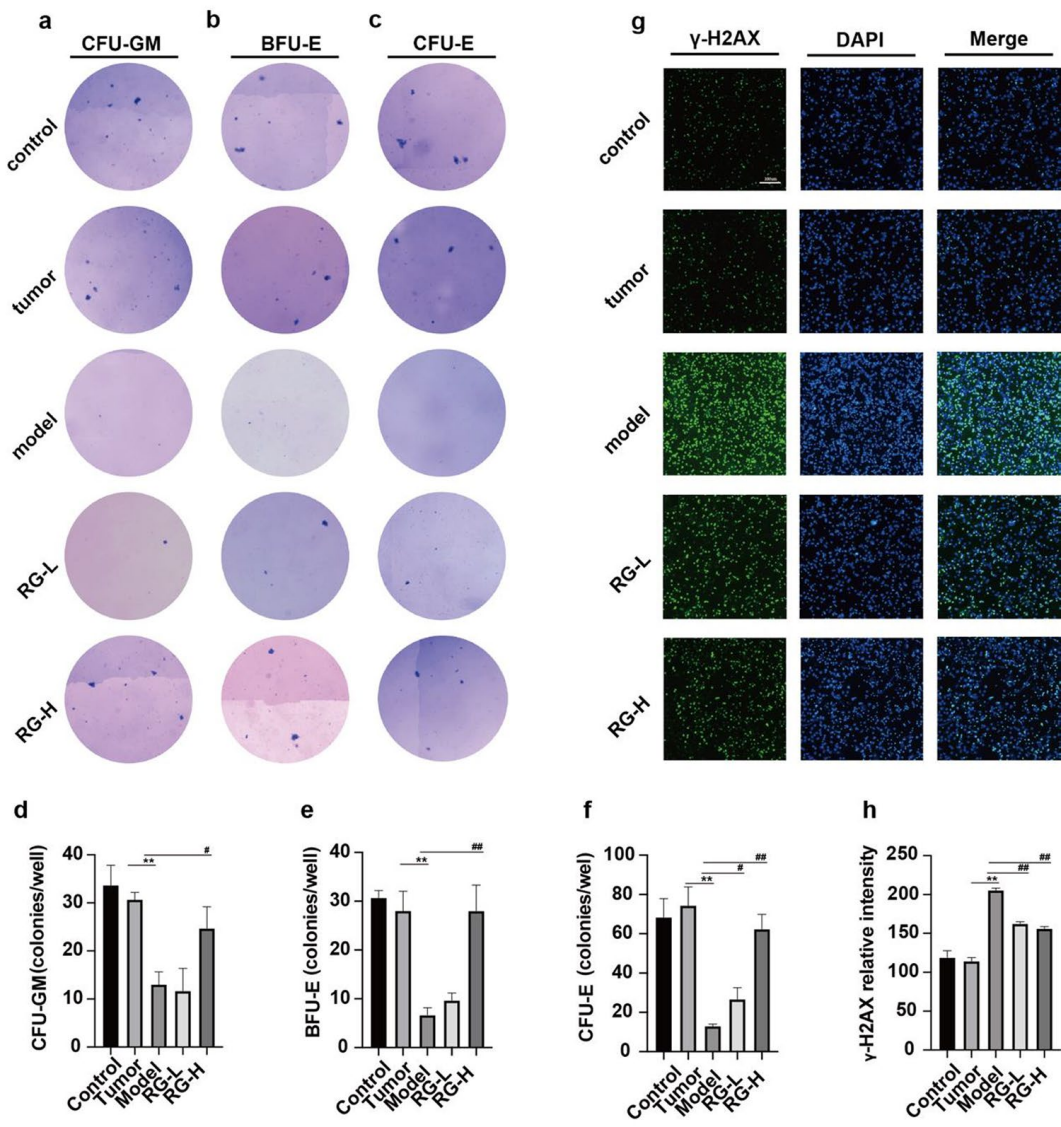
Effect of RG on hematopoietic progenitor cells and the expression of γ-H2AX, a DSBs marker in niraparib-induced mice. The Wright–Giemsa staining of colony yields (**a**–**c**); the yields of CFU-GM, BFU-E, and CFU-E colonies (**d**–**f**); the expression levels of γ-H2AX (**g**); the expression of γ-H2AX detected by immunofluorescence (**h**). The images were captured at a magnification of ×400. The scale bar indicated in the control group is 20 µm; values are expressed as mean ± SD (n = 6). Statistical significance was taken at **p < 0.01 against the tumor group. ^#^*p* < 0.05 and ^##^*p* < 0.01 against the model group

HPCs, which ultimately differentiate into mature blood cells, play an important role in maintaining uninterrupted hematopoiesis [10]. As shown in Fig. 3a–f, the results showed that, compared with the control and tumor groups, the yields of the CFU-GM, BFU-E, and CFU-E colonies cultured in vitro after niraparib administration decreased. RG-L treatment only increased the CFU-E yield, while RG-H treatment significantly increased the yields of CFU-E, BFU-E, and CFU-GM, indicating that RG probably improves HPC reserves and protects HPC self-renewal.

### Effect of RG on DSBs

PARP1 trapping by PARP inhibitors drives cytotoxicity in healthy bone marrow [11]. When DNA damage occurs in the presence of niraparib, PARP1 binds to damaged sites and progresses DNA DSBs. Phosphorylation of histone H2AX at serine 139 is sensitive to binding with DSBs. As shown in Fig. 3g–h, niraparib significantly increased γ-H2AX positive staining in BMNCs compared with the control and tumor groups. As expected, both RG-L and RG-H treatments markedly reduced the expression level of γ-H2AX. These results demonstrate that RG could reduce the DNA damage of BMNCs.

### Effect of RG on cell cycles

DNA damage initiates cell cycle arrest until the damaged DNA is repaired [12]. As shown in Fig. 3a, niraparib treatment caused a significant increase in the percentage of cells in the G0/G1 phase, and decreased the PI compared with the control and tumor groups. Both RG-L and RG-H treatment decreased the percentage of cells in the G0/G1 phase (*p* < 0.01) and increased the PI (*p* < 0.01). These findings support that RG could improve G1 phase cell cycle arrest.

### Effect of RG on the expression levels of p53 pathway-related proteins in BMNCs

**Fig. 4:**
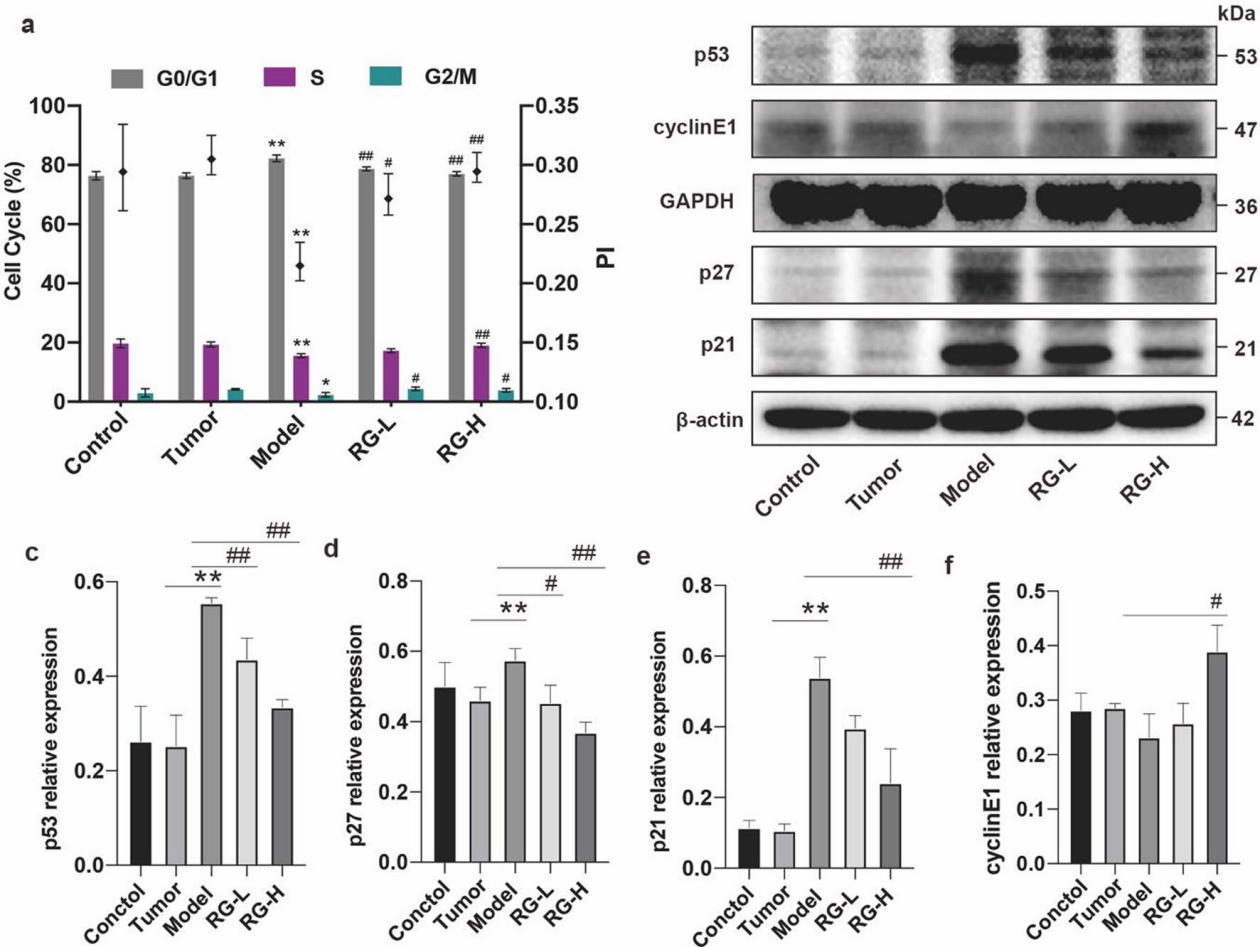
Effect of RG on cell cycle and p53 pathway-related proteins in niraparib-induced mice. The cell cycle of different groups (**a**); the expression of p53, p27, p21, CyclinE1, and β-actin proteins was evaluated by western blot (**b**); the relative expression of p53/GAPDH, p27/β-actin, p21/β-actin, and CyclinE1/GAPDH (**c**–**f**). Values are expressed as mean ± SD (n = 3). Statistical significance was taken at *p < 0.05 and **p < 0.01 against the tumor group, ^#^*p* < 0.05 and ^##^*p* < 0.01 against the model group

The critical role of the p53 pathway in cell cycle arrest in response to DNA damage from various stressors is well-known [13]. In comparison to the control and tumor groups, the model group exhibited significantly increased expression levels of p53, p21, and p27. RG-L and RG-H treatment reduced the expression levels of p53 and p27. Only RG-H treatment significantly reduced the expression level of p21 and increased cyclinE1 (Fig. 4b–e). The effects of RG-H were more pronounced than those of RG-L on these protein expression levels. These results suggest that RG may alleviate niraparib-induced cell cycle arrest by inhibiting p53-mediated upregulation of p21 and p27.

### Effect of RG on tumor growth in niraparib-induced mice

**Fig. 5:**
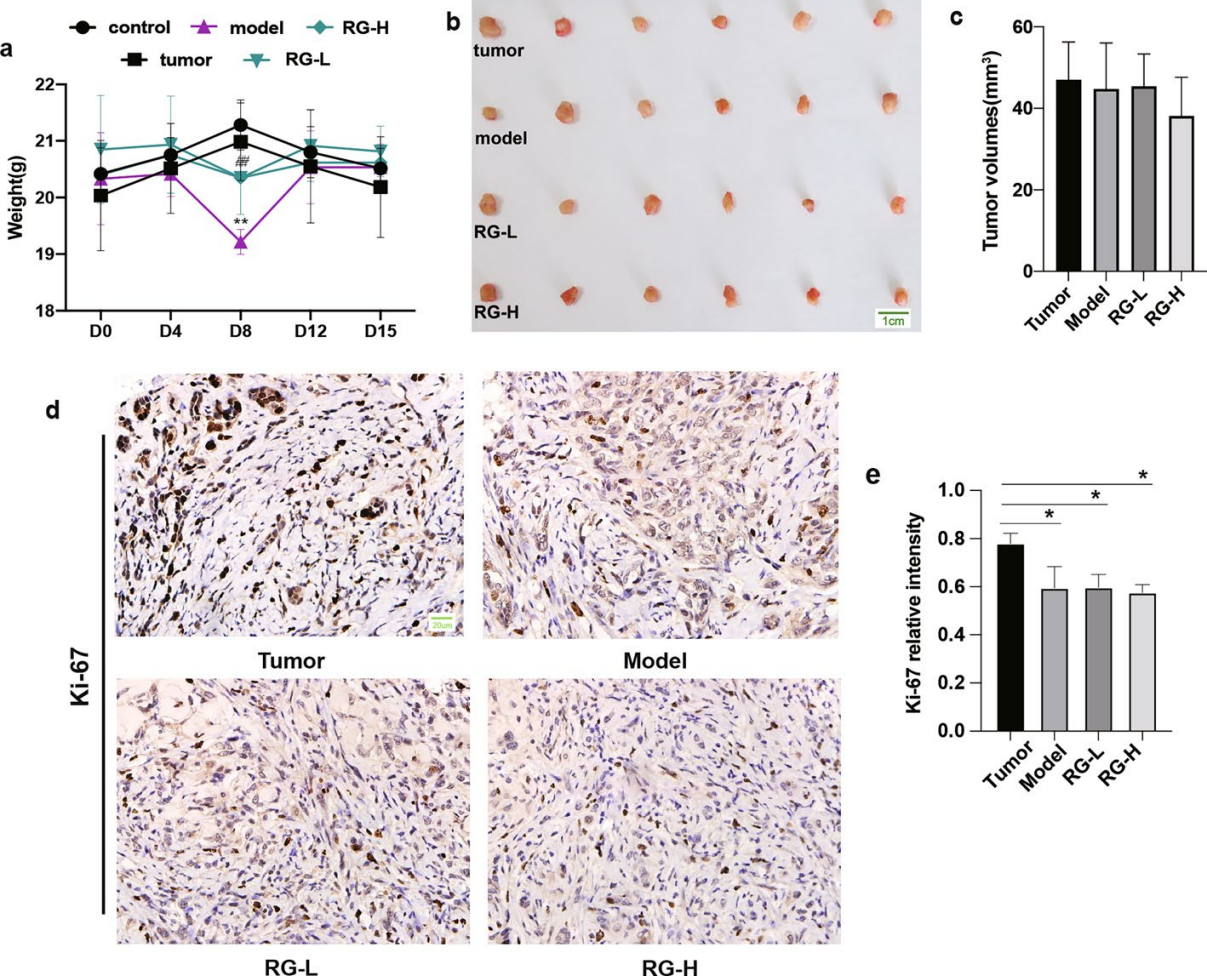
Effect of RG on body weight and tumor growth. Body weights of mice (**a**). Values are expressed as mean ± SD (n = 6). Statistical significance was taken at **p < 0.01 against the tumor group, ^##^*p* < 0.01 against the model group. Tumor volumes of mice in different groups (**b**). Statistical results of tumor volumes (**c**). The immunohistochemical images showing Ki-67 staining (**d**). The images were captured at a magnification of 400 × . The scale bar indicated in the control group is 20 µm. The expression levels of Ki-67 (**e**). Values are expressed as mean ± SD (n = 3), *p < 0.05 compared with the tumor group

To investigate the safety of RG, we assessed the physical activity of mice and the anti-tumor effects of niraparib in combination with RG. As shown in Fig. 5a, niraparib significantly reduced the weight of the mice on day 8. In contrast, RG treatment ameliorated the weight loss caused by niraparib. RG did not increase tumor volume (Fig. 5b–c). Compared with the control and tumor groups, the expression level of Ki-67 significantly decreased in the model group. The expression level of Ki-67 in the RG-L and RG-H groups was similar to that in the model group (Fig. 5d–e). These findings suggest that RG does not harm the anti-tumor activity of niraparib.

## Discussion

In Asian countries, natural medicines are often used in combination with chemotherapy or radiotherapy to prevent or reduce their side effects and complications, while reducing treatment costs [14–16]. However, few studies have focused on whether these natural medicines can be combined with targeted drugs to prevent the occurrence of myelosuppression. Unlike short-term chemotherapy, the PARP inhibitor, niraparib, a targeted drug, is commonly used for long-term maintenance therapy. However, myelosuppression induced by PARP inhibitors may lead to the reduction, suspension, or even discontinuation of the drug. This not only impacts patient survival and prognosis, but also reduces quality of life [17]. Therefore, preventing and reducing myelosuppression to ensure long-term safe use of PARP inhibitors is of great importance. This study is the first to explore the effects and potential mechanisms of RG in niraparib-induced myelosuppression.

Ginseng contains various components such as ginsenosides, polysaccharides, volatile oils, and nitrogen-containing compounds [16]. Polysaccharides of ginseng can promote the exposure of small molecules and increase their bioavailability, which enhances drug efficacy [14]. What is more impressive is that ginsenosides are considered the main pharmacological active ingredient of ginseng and improve bone marrow suppression in mice [16]. Regarding the chemical profiles of RG, the major ginsenosides in RG from our study were consistent with other reports [18]. Among the nine ginsenoside monomers identified from the UHPLC-QE-MS analysis, some play an active role in relieving chemotherapy-induced myelosuppression. For instance, ginsenoside Rg1 is effective in reducing the decline in BMNCs, elevating the number of peripheral blood cells, and enhancing the recovery of hematopoiesis [18]. In addition, Rg1 could improve the migration of hematopoietic stem and progenitor cells from the spleen to the bone marrow [19]. Ginsenoside Re has been shown to ameliorate CTX-induced myelosuppression by reducing the ratio of G0/G1 phase cells and increasing the PI [20]. Ginsenoside Rb1 has a significant protective effect against DNA damage and cell apoptosis induced by cyclophosphamide [21]. These active ingredients not only confirm the pharmacological activities of RG and its positive effects on bone marrow, but also provide a theoretical basis for addressing niraparib-induced myelosuppression.

Niraparib traps PARP1/2 in DNA at sites of single-strand breaks, thereby preventing the repair of these breaks and generating DSBs [22]. In this study, the expression level of γ-H2AX was significantly increased by niraparib, indicating that niraparib administration led to the accumulation of DSBs in BMNCs. DSBs caused by niraparib may activate signaling molecules and result in cell cycle arrest. A high ratio of cells in the G0/G1 phase after niraparib exposure resulted in decreased cell viability. However, the reduction in hematopoietic effect caused by niraparib can be significantly mitigated by RG treatment, reducing the expression level of γ-H2AX and facilitating the repair of damaged DNA. With an increased PI, RG promoted more cells passing the G1 phase checkpoint to enter the S phase for DNA synthesis and then enter the G2/M phase for completion of mitosis. These findings indicate that RG can exert a protective effect against cell cycle arrest, which may reduce the number of DSBs. Consequently, RG demonstrated a positive impact on enhancing the survival of damaged cells, effectively alleviating the reduction in the number of BMNCs and the yield of HPC colonies caused by niraparib. The expression level of p53 in bone marrow cells increases following DSBs [23]. According to our study, RG treatment effectively suppressed the expression levels of p53, p21, and p27, while simultaneously stimulating the expression levels of cyclinE1, ultimately alleviating the cell cycle arrest. This, in turn, promoted the restoration of bone marrow hematopoietic function.

This study also indicated that RG had no impact on body weight in mice. Ki-67, a pro-proliferative marker [24], was suppressed by niraparib, suggesting that niraparib treatment is effective in inhibiting tumor growth. Additionally, neither RG-L nor RG-H showed increased Ki-67 expression level. Overall, RG was proven to be a safe agent that did not affect the anti-tumor activity of niraparib.

In summary, our findings indicated for the first time that RG ameliorated niraparib-induced myelosuppression by facilitating DNA damage repair and alleviating cell cycle arrest. The protection effect of RG was associated with the regulation of p53-mediated upregulation of p21 and p27. RG may be a promising drug to attenuate niraparib-induced myelosuppression. Further studies are required to evaluate the effects on the protection of hematopoietic stem cells.

## Abbreviations

BMNC: Bone marrow nucleated cell;
BFU-E: Burst-forming unit-erythroid
CFU- E: Colony-forming unit-erythroid
CFU-GM: Colony-forming unit-granulocyte–macrophage
HgB: Hemoglobin
HPC: Hematopoietic progenitor cell
NEU: Neutrophil granulocytes
PLT: Platelets
WBC: White blood cells
